# Editorial: Saving mothers and babies for the new world

**DOI:** 10.3389/fsurg.2023.1291596

**Published:** 2023-09-29

**Authors:** Azanna Ahmad Kamar, Fook-Choe Cheah, Hamizah Ismail, Ranjan Pejaver, David Alan Ellwood, Zaleha Abdullah Mahdy

**Affiliations:** ^1^ Neonatal Intensive Care Unit, Department of Paediatrics, Universiti Malaya, Kuala Lumpur, Malaysia; ^2^ Neonatal Intensive Care Unit, Department of Paediatrics, National University of Malaysia, Kuala Lumpur, Malaysia; ^3^ Department of Obstetrics & Gynaecology, International Islamic University, Kuantan, Malaysia; ^4^ Neonatology Unit, People Tree at Meenakshi Hospitals, Bangalore, India; ^5^ School of Medicine and Dentistry, Griffith University, Birtinya, QLD, Australia; ^6^ Department of Obstetrics and Gynecology, Faculty of Medicine, National University of Malaysia, Kuala Lumpur, Malaysia

**Keywords:** perinatal care, obstetrics, neonatology, fetal, perinatal mortality

**Editorial on the Research Topic** Saving mothers and babies for the new world

## Introduction

First, where or what is the *New World*? Historically, this refers to the region of the Americas. In this context, the *New World* is the future of humankind, an ambiguous borderless phenomenon that is informed and built upon from lessons of its past and from stories of triumph and regrets; of innovations, experiences of the pandemic, economic successes, and failures; of those unwanted wars; and of great science. The *New World* we envision emphasizes the quintessential of actions to ensure human survivability, by prioritizing equitable care to mothers and babies regardless of their region. Positive outcomes for mothers and babies can only be achieved by ensuring that healthcare professionals are knowledgeable, holistic, ethical, and safe and that effective execution of ethically robust healthcare policies and guidelines for equitable perinatal healthcare is adequately supported. These aims need to be at the forefront of policies of governments across all countries in the *New World* despite all setbacks. With this in mind, the research topic *Saving Mothers and Babies for the New World* accepted 16 diverse full-length articles and collected abstracts presented at the 21st Federation of Asia Oceania Perinatal Societies (FAOPS) Congress that provided a wealth of information and science that can help carve future research and inform policies.

## The crystal ball: accurate data can help predict adverse perinatal outcomes

The rich diversity found in large databases from developing countries can help predict various pregnancy-related problems. Hypertension is a leading medical problem encountered during pregnancy with profound effects to both the mother and her baby. Zhang et al. appraised the accuracy of the LASSO regression dynamic prediction model in predicting the four subtypes of hypertensive disorder in pregnancy (HDP). The predictive capability was above 85%, with the highest being close to 92%, accurately predicting the risk of these four subtypes. In the review of 13 studies by Sukor et al. they concluded that endothelial function was impaired in the offspring of women with *de novo* HDP. The need to study and monitor the cardiovascular health of these affected infants, whether they are predisposed to hypertension or ischemic heart disease in later life, brings to attention of neonatologists. Some complexities in monitoring the fetus during antenatal care (ANC) was shared by Saw et al. who discovered that using the Hadlock and INTERGROWTH-21 chart in the Malaysian population may result in misdiagnoses of fetuses that are small for gestational age (SGA). Moreover, a differential accuracy in predicting preterm SGA according to trimester, with poorer accuracy in the second trimester, is found. Qiu et al. demonstrated that microtia is the subject to scrutiny. It is often accompanied by congenital defects of other organs and structures, especially the heart and face. Hence, prenatal diagnosis of microtia and associated anomalies by ultrasound is of important clinical significance.

## Teamwork and safety: collaborative management and safe perinatal interventions

Two case reports in this collection further highlight the importance of concerted perinatal efforts to achieve better outcomes. Sun et al. described an interesting case management of parturients with acute myocardial infarction resulting in positive successful outcomes for both the mother and the fetus through multidisciplinary team management, consisting of obstetricians, cardiologists, anesthetists, and pediatricians. Yantao et al. narrated a pregnancy complication called Meckel's diverticulum, which is a condition that is not easily diagnosed. Once it is highly suspected, especially with peritonitis, surgical emergency helps save the life of the mother and the baby. In this special issue, reports on techniques that improve perioperative care and therapeutic advances in reproductive care include a randomized comparative trial conducted by Pang et al. This group reported that epidural dexmedetomidine was a better alternative to standard-dose epidural fentanyl in reducing the mean hourly amount of ropivacaine administered and minimizing opioid-related side effects in women who needed labor analgesia. Regarding postpartum care, Lizheng Zhao and Hong Wei recommended to strengthen the Enhanced Recovery After Surgery (ERAS) guideline and cooperation among researchers in order to generate a broader consensus and results and ultimately provide help for cesarean section recovery. An interesting observation by **Huang et al.** concentrated on the relationship between cervical length (CL) and massive intraoperative bleeding in patients with placenta accreta spectrum (PAS). They found that when the CL was greater than 33 mm, the risk of bleeding decreased by 44%. Therefore, CL is capable of functioning as a standalone parameter to identify the risk of massive intraoperative bleeding during cesarean section in patients with suspected PAS. Within the community in the Tibetan Plateau region, Long et al. found that vaginal delivery for term breech in the lithotomy position was less safe than cephalic presentation. Timely recognition of problems and availability of cesarean section greatly improved the safety.

## Back to basics, be kind: good perinatal care and zero abuse required

Proper and effective perinatal care, from fertility preservation to conception and careful ANC, is crucial in ensuring a healthy pregnancy outcome. **Han et al.** questioned whether the function of reproductive organs of a woman with adenomyosis can be preserved and looked into the treatment of adenomyosis in relation to infertility. The authors advised that an individualized strategy based on the grading and needs of the patient is required to achieve pregnancy. **Chilot et al.** lamented on the relatively low frequency of optimal ANC utilization in countries with high maternal mortality. It was found that both individual-level factors and community-level factors were significantly associated with ANC utilization. This provided appropriate guidance and basis for targeted prevention of adverse outcomes with improved care. The emphasis on not only physical health of the woman but also protection of their mental health, as well as individualizing treatment, is certainly timely, in order to achieve a satisfactory reproductive outcome. In this context, Gebeyehu et al. found a high prevalence of disrespect and abuse of women during childbirth in East Africa. Predictors of maternal disrespect and abuse include instrumental delivery, childbirth complications, receiving care at government hospitals and a poor wealth index.

## Strategize: preventing preterm births and neonatal deaths

The rate of preterm births has not improved in the past decade, and prematurity remains as one of the leading causes of perinatal and neonatal deaths. According to the World Health Organization (WHO), this is translated to an estimated 13.4 million babies born preterm in 2020 with nearly 1 million dying from complications related to prematurity. This is an area to focus on if we are to achieve the sustainable development goal (SDG) target 3.2 that aims to prevent deaths of newborns and children under 5 years of age by 2030. Sub-Saharan Africa is one of the regions with the highest number of preterm births. A retrospective follow-up study of Girma et al. in Ethiopia reported that nearly one in three preterm neonates (32.1%) will die with a mean survival time of 18.7 days. The two major predictors of death are respiratory distress syndrome and perinatal asphyxia. On a positive note, preterm infants who received kangaroo mother care (KMC) are much less likely to die. In mitigating the high mortality rates of preterm infants in low- and middle-income countries (LMIC), KMC or skin-to-skin contact nursing may be a solution. Previously, KMC has been targeted to caring of the stable-growing preterm infant. However, the WHO has recently launched new guidelines (November 2022) for immediate kangaroo mother care (iKMC) to improve survival of infants born preterm and having low birth weight. Infection during pregnancy is a recognized cause of preterm birth. Bacterial vaginosis (BV) has been reported as a risk factor for preterm labor although not many extensive studies have been conducted especially from the LMIC and study methods were variable. Ng et al. used a rapid point-of-care test based on a chromogenic response to increased sialidase in vaginal swab samples and detected one in 10 pregnant women to have BV, with variable rates depending on the method of detection used and the presenting symptoms. On this note, the authors highlighted that BV could be much higher (one in four or five) among women with preterm labor as was the case in two other studies they cited. Preterm birth below 34 weeks was almost four times more likely to be associated with BV. Consequently, neonatal morbidity was greater with twice more likely admissions to the neonatal intensive care unit because of respiratory problem requiring support. All the BV-positive pregnancies were treated with vaginal pessaries containing dequalinium chloride, which has a broad antimicrobial spectrum. Although the intervention did not appear to prolong the gestational period of the pregnancy to term, future research for this condition may provide us insights of its role in reducing preterm births.

## Keep it going: ensuring sustainability of good practices

Positive outcomes require sustainability of good practices. In this issue, **Xu et al.** shared their model of multi-disciplinary *in situ* simulation training (MIST) in Shenzhen, China. Collaboration between neonatal and obstetric healthcare providers to conduct a weekly simulation training exhibited a significant reduction in neonatal asphyxia. More than one-third of the sessions included resuscitation of preterm neonates. Implementing such regular on-site simulation training should be considered in countries that encounter high rates of mortality in asphyxiated preterm infants. It is imperative that neonatal resuscitation and stabilization competency through workshops or courses such as Helping Babies Breathe, Neonatal Resuscitation Program (NRP), and S.T.A.B.L.E. are intensified in a global sense with pooling of resources and funding for training of personnel and basic infrastructure. The sustainability of such programs and the integration of simulation healthcare are much needed to ensure that health staff remain competent in saving lives.

## The new world

The *New World* is right at our doorstep. Reducing maternal deaths and ensuring survival of even the littlest infant demand collective effort, not only from the perinatal health fraternity but also from every individual within. To save mothers and babies, we need accurate data, perform safe collaborative teamwork, practice quality perinatal care, launch effective preventive strategies, and ensure that all these practices are sustained for the future and the *New World*.

*As mankind weeps,*

*Therein lies the embrace,*

*Of the mother and her baby,*

*Where saving is our duty,*

*For the new world's gates.*

